# Infection Spread, Recovery, and Fatality from Coronavirus in Different Provinces of Saudi Arabia

**DOI:** 10.3390/healthcare9080931

**Published:** 2021-07-24

**Authors:** Mohammed Muberek Alharbi, Syed Imam Rabbani, Syed Mohammed Basheeruddin Asdaq, Abdulhakeem S. Alamri, Walaa F. Alsanie, Majid Alhomrani, Yahya Mohzari, Ahmed Alrashed, Reem Faisal Bamogaddam, Saleh Ahmad Alajlan, Mansour A. Alharbi, Norah N. Aldhawyan, Saeed A. Najmi

**Affiliations:** 1 Department of Pharmacology and Toxicology, College of Pharmacy, Qassim University, Buraydah 51452, Saudi Arabia; mhospital1920@gmail.com (M.M.A.); syedrabbani09@yahoo.com (S.I.R.); 2 Department of Pharmacy Practice, College of Pharmacy, AlMaarefa University, Dariyah, Riyadh 13713, Saudi Arabia; 3 Department of Clinical Laboratory Sciences, The Faculty of Applied Medical Sciences, Taif University, Taif 21974, Saudi Arabia; a.alamri@tu.edu.sa (A.S.A.); w.alsanie@tu.edu.sa (W.F.A.); m.alhomrani@tu.edu.sa (M.A.); 4 Centre of Biomedical Sciences Research (CBSR), Deanship of Scientific Research, Taif University, Taif 21974, Saudi Arabia; 5 Clinical Pharmacy Department, King Saud Medical City, Riyadh 12746, Saudi Arabia; Yali2016@hotmail.com (Y.M.); manalharbi87@gmail.com (M.A.A.); Ph.norahnd@gmail.com (N.N.A.); 6 Pharmaceutical Services Administration, Inpatient Department, Main Hospital, KFMC, Riyadh 11525, Saudi Arabia; emadasdaq@gmail.com (A.A.); qualityasdaq@gmail.com (S.A.A.); 7 Pharmaceutical Services Department, Clinical Pharmacy, King Saud Medical City, Riyadh 12746, Saudi Arabia; farhana.basheer13@gmail.com; 8 Pharmaceutical Care Services, Samtah General Services, Ministry of Health, Jazan 86735, Saudi Arabia; sanajmi-2017@hotmail.com

**Keywords:** COVID-19, pattern of spread, recovery, mortality, provinces, Saudi Arabia

## Abstract

The World Health Organization (WHO) announced COVID-19, a novel coronavirus outbreak, as a pandemic in 2020. In the month of February 2020, the disease began to spread through the Middle East. The first case of COVID-19 in the Kingdom of Saudi Arabia was identified in March 2020, and it is now one of the region’s most affected countries. Analyzing the disease’s propagation pattern may aid in the development of pandemic-fighting strategies. This study aims to analyze the trend of COVID-19’s spread, its recovery, and mortality in the Kingdom of Saudi Arabia (KSA). Two to three major cities from the 13 provinces of the country were chosen, and the rate of infection recovery was recorded from the first month until the number of confirmed cases showed a decline. The data published on the official Ministry of Health website were recorded on an Excel sheet, graphically represented as figures to indicate the pattern of spread. According to the study’s findings, COVID-19 positive cases were discovered in the majority of provinces as early as March 2020. The province of Makkah had the largest number of COVID-19 positive cases (30.7%), followed by Riyadh (23%). The province of Al Jowf had the lowest number of COVID-19 cases (0.3%). Tabuk province had the highest rate of recovery (97.8%), followed by the Northern Border Province (96.7%). Makkah province had the highest mortality rate (2.6%), followed by Al Jawf province (2.4%). The peak case–fatality ratio was recorded in August and September. The highest number of tests to detect the COVID-19 was performed in the month of July, and the highest percentage of positive cases was detected in June (19.55%). All the provinces from the month of September 2020 showed a progressive decline in the number of confirmed COVID-19 cases. According to this study, COVID-19 infection was found in the majority of Saudi Arabian provinces in March 2020, with a peak in June–July 2020. Considering the climatic and demographic characteristics of the region, specific modalities need to be adopted in collaboration with international guidelines to defeat the COVID-19 pandemic.

## 1. Introduction

COVID-19 is a highly infectious illness caused by a serious acute coronavirus respiratory syndrome [1]. The outbreak of COVID-19 disease 2019 started in Wuhan province in China; the epidemic is more widespread than initially estimated, with cases now confirmed throughout the world [2].

Many initial cases of COVID-19 were related to the Huanan Seafood wholesale market, implying that SARS-CoV-2 was transferred from animals to humans [3]. As the outbreak progressed, human to human became the main mode of transmission. The incubation period for SARS-CoV-2 is within 14 days after exposure, but most cases show symptoms within 4 to 5 days of exposure [4].

COVID-19 can cause illnesses ranging from the common cold to more severe diseases such as Middle East Respiratory Syndrome (MERS-CoV) and Severe Acute Respiratory Syndrome (SARS-CoV-2) [5]. According to the WHO, as of 1 May 2020, there have been more than 5 million confirmed cases of COVID-19 around the world. The latest data indicate that the number has crossed 50 million confirmed cases of COVID-19. The daily report of confirmed cases can be accessed at the link http://Covid19.cdc.gov.sa (accessed on 13 July 2021). The current methods of diagnosis of COVID-19 include detection of the virus or viral components by genomic techniques using either polymerase chain reaction (PCR)-based methods or deep sequencing [6,7,8].

According to the literature, all individuals are at risk of acquiring COVID-19 [9]. Male patients and those with other chronic comorbid conditions such as respiratory and cardiovascular diseases are more likely to die as a result of SARS-CoV-2 [10,11,12].

All pandemics follow a certain pattern of population distribution. Understanding this could enable policymakers to assess the path of infection, which could aid in the creation of pandemic-control strategies. Saudi Arabia is mostly comprised of deserts with some forests, grasslands, and mountains. Less than 7% of the land is suitable for cultivation. The population of the country is mostly located in the eastern and western coastal areas and rarely in deserts. The country has 13 provinces, and the population is mainly pocketed in cities and small towns [13,14]. Since the pattern of infectious disease is influenced by several factors, such as urbanization, socio-economic, infrastructure, and educational qualifications, this study focused on the spread, recovery, and mortality from the ‘first wave’ of COVID-19 in major cities of different provinces of Saudi Arabia.

## 2. Materials and Methods

### 2.1. Study Area

A retrospective analysis of the spread of COVID-19 in different provinces of the Kingdom of Saudi Arabia was performed. The country consists of vast deserts, and the population is remotely distributed into separate regions, called provinces. There are 13 provinces, Riyadh, Makkah, Eastern, Al Madinah, Al Qassim, Hail, Northern border, Asir, Al Baha, Jazan, Al Jawf, Najran, and Tabuk, which include several cities, towns, and villages. In this study, two to three major cities in each province were selected to evaluate the pattern of spread, recovery, and mortality rate [15].

### 2.2. Collection and Recording of Data:

The official data published by the Ministry of Health (MoH), Saudi Arabia, was used to record the number of cases daily (both symptomatic and asymptomatic) starting from March (https://Covid19.cdc.gov.sa/daily-updates/ accessed on 13 July 2021). The data about the number of cases reported in the selected two-three major cities were recorded in an Excel file. The recording was performed from the month when the first case was reported in this region until October 2020. This period was considered to be the ‘first wave’ of COVID-19 in the country. From October 2020, the region witnessed a significant decline in the number of COVID-19 cases.

### 2.3. Analysis of Data

The data collected from day one were entered into an Excel spreadsheet. The values were expressed separately for each province to demonstrate the trend of the pandemic spread in different months. The following parameters were analyzed to study the infection spread, recovery, and fatality from COVID-19.

#### 2.3.1. Pattern of Spread of COVID-19 in Different Provinces

The data was collected from the first day when a confirmed COVID-19 positive case was officially detected in the country. The confirmed numbers of cases reported by the Ministry of Health were recorded for every province per month.

#### 2.3.2. Percentage of Confirmed Cases Detected

This parameter determines the total number of COVID-19 tests conducted in a month and the percentage of confirmed cases detected.

#### 2.3.3. Infection Rate in Population of Different Provinces

The infection rate was calculated for each month using Formula [16]: Number of confirmed cases × 100/Total number of cases.

#### 2.3.4. Rate of Recovery from COVID-19 in Different Provinces

The daily reported cases of recovery from different provinces were used to calculate the rate of recovery.

#### 2.3.5. Mortality Rate from COVID-19 in Different Provinces

The official number of deaths reported from different provinces was used to record the mortality rate.

#### 2.3.6. Case-Fatality Rate (CFR) in Different Provinces

The CFR was calculated by using a formula used by Onder et al., 2020 [17].

CFR= (Total number of deaths × 100)/Total number of cases.

#### 2.3.7. Total Pattern of Spread of COVID-19 in Different Provinces

The total number of tests conducted, confirmed cases detected, recoveries, and deaths reported from different provinces was summarized for every month to determine the pattern of the spread of COVID-19 in Saudi Arabia.

### 2.4. Representation of Data

The data were represented graphically in the form of figures to indicate the number of cases reported in different months as well as the infection rate, percentage of confirmed cases detected, recovery rate, mortality rate, and case-mortality ratio in Saudi Arabia.

### 2.5. Statistics

The data on the confirmed COVID-19 cases and the number of recoveries and deaths were recorded for each province. A descriptive analysis was performed on the collected data. Each month’s values were separated. The GraphPad Prism 8.0 computer software package was used to summarize the data and plot the graphs. The *X*-axis in the figure indicates the months, while the *Y*-axis represents the number of confirmed cases/infection rate/number of recovery/confirmed deaths.

## 3. Results

### 3.1. Pattern of Spread of COVID-19 in Different Provinces of Saudi Arabia

**Figure 1:**
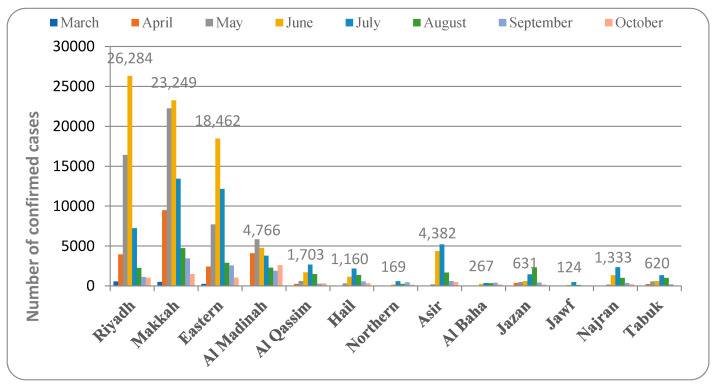
Pattern of spread of COVID-19 in different provinces of Saudi Arabia.

Figure 1 depicts the pattern of COVID-19 distribution in various provinces. Riyadh province recorded cases beginning in March 2020. The peak increase in cases was detected in June 2020, and from then on, the number of confirmed cases started to decline. The total percentage of COVID-19 detected in this province was 23%. The pattern of spread of COVID-19 in Makkah province was active in March 2020, increasing in April and May and reaching a peak in June, then showing a slow decline (30.7% of total cases). Similarly, in the Eastern province, the first case was detected in March 2020 and the number peaked in June and then declined. The spread of COVID-19 started in the month of March 2020 and peaked in May and then showed a slow decrease in the number of confirmed cases (18.6% of total cases).

The Al Madinah province detected the first case in March 2020 and the rate of infection peaked in May (9.9% of total cases). In Al Qassim province, the month of March 2020 reported the first case, and infection increased in April, May, and June and reached a peak in July (2.8% of total cases). In Hail province, the spread of COVID-19 started in the month of April and reached a peak in July, then decreased slowly (2.3% of total infection). The northern border province recorded the first case in March 2020, and the infection increased, reaching its peak in July (0.7% of the total infection). Asir province reported the first case in March 2020 and then peaked in July (4.9% of total confirmed cases).

In Al Baha province, the pattern of spread of COVID-19 was found in March 2020, the cases peaked in July, and the following months showed a gradual decline in the rate of infection (0.6% of total infection). The Jazan province data indicated that the first positive case was detected in March 2020, which spread fast to reach a peak in August, and then the spread declined slowly (2.3% of total infection). The Al Jawf province recorded its first COVID-19 case in the month of April, which later spread, reaching its peak in July (0.3% of the total infection detected). Positive COVID-19 cases in Najran province were detected in March 2020, and the infection was found to have a peak in July (2.1% of infection). Tabuk province recorded a COVID-19 active case in March 2020. The infection spread to other parts of the region, peaking in July (1.6% of the total infection).

### 3.2. Percentage of Confirmed COVID-19 Cases Detected

**Figure 2:**
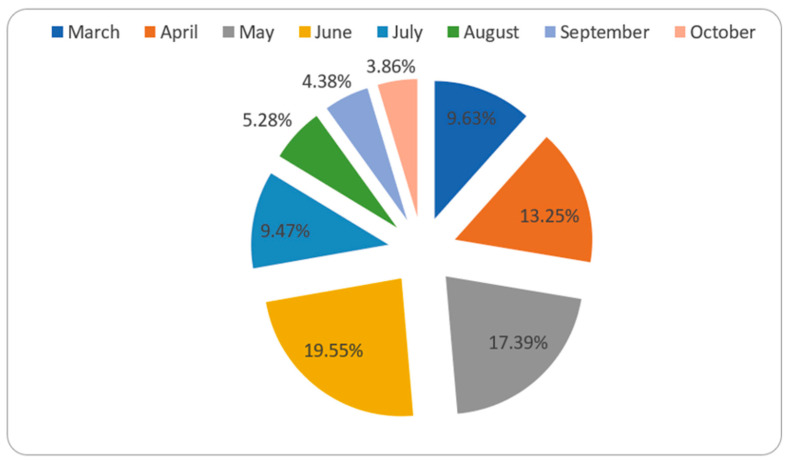
Percentage of confirmed cases detected in each month.

The first case of COVID-19 was discovered in March, and the percentage of confirmed COVID-19 cases calculated from the total number of tests in this month was 9.63%. This rate increased faster and reached its peak in June (19.55%). The rate then began to fall, and extensive testing was conducted, yielding the lowest detected percentage of confirmed cases (3.86%) (Figure 2).

### 3.3. Infection Rate in Population of Different Provinces of Saudi Arabia

**Figure 3:**
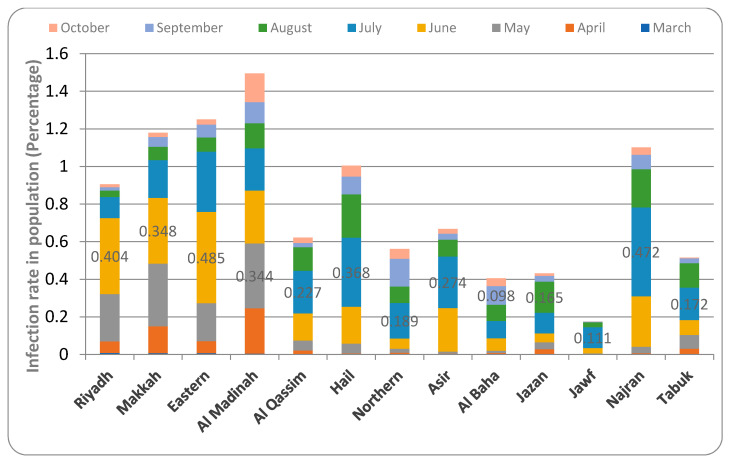
Infection rate in population of different provinces of Saudi Arabia.

The rate of infection in Saudi Arabia indicated that all the provinces recorded COVID-19 cases in the month of March except Hail and Jawf. Riyadh province had the highest infection rate (0.008% of the population) in this month, followed by Makkah province (0.007%), Eastern province (0.006%), and Al-Qassim (0.0004%). The infection rate gradually increased and reached peaks in the month of May (Al Madinah—0.344%), June (Riyadh—0.404%, Makkah—0.348%, Eastern—0.485%), July (Al-Qassim—0.227%, Hail—0.368%, Northern borders—0.186%, Asir—0.274%, Jawf—0.111%, Najran—0.472% and Tabuk—0.172%), August (Jazan—0.165%), and September (Al Baha—0.098%). Since these months, the COVID-19 infection rate has gradually decreased in all provinces of the country. Al Madinah province had the highest infection rate (0.15%) in October, followed by Hail (0.05%), Najran (0.03%), and Jawf (0.0007%) (Figure 3).

### 3.4. Rate of Recovery from COVID-19 in Different Provinces of Saudi Arabia

**Figure 4:**
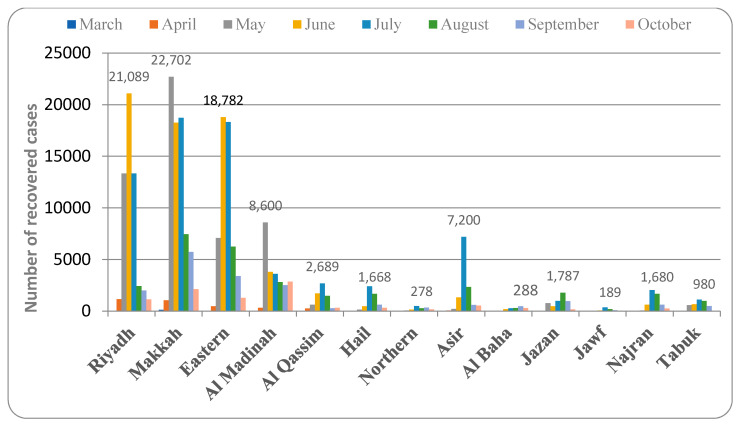
Rate of recovery from COVID-19 in different provinces of Saudi Arabia.

According to the available data, the rate of recovery in Riyadh province increased gradually beginning in March 2020 and peaked in June (95.6% recovery). In Makkah province, the rate of recovery started to increase in March 2020 and peaked in May (96.4% recovery). The eastern province showed recovery from COVID-19 from March 2020, and the peak rate was observed in June (95.9% recovery). In Al Madinah province, the peak rate of recovery was found in May, though the province showed this in the month of March 2020 (96.7% recovery). Al Qassim province showed a recovery from March 2020, and the peak rate was found in July and then slowly declined (95.8% recovery). In Hail province, the peak rate of recovery from COVID-19 was found in July (94.2% recovery). Similarly, the peak rate of recovery was found to be in the month of July in the Northern border (96.7% recovery) and Asir (96.3% recovery) provinces. In Al Baha province, the peak rate of recovery was found in the month of September (95.4% recovery), while in Jazan, it was in August (96.6% recovery). Al Jawf (95.5% recovery) and Najran (96.3% recovery) provinces showed a peak rate of recovery in the month of July. The maximum recovery from the COVID-19 vaccine in Tabuk province was found in both the July and August months, and the total recovery was found to be 97.8% (Figure 4).

### 3.5. Mortality Rate from COVID-19 in Different Provinces of Saudi Arabia

**Figure 5:**
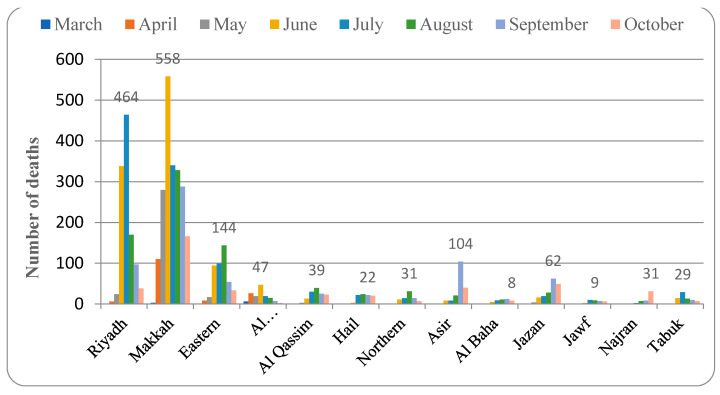
Mortality rate from COVID-19 in different provinces of Saudi Arabia.

The data for the mortality rate from COVID-19 infection are summarized in Figure 5. Riyadh province reported mortality in the month of March 2020, and this peaked in July (total mortality rate of 1.9%). Makkah province followed a similar pattern, with mortality beginning in March but peaking in June (mortality rate 2.6%). The mortality in the Eastern province was found in April and reported its peak death rate in August (mortality rate of 1.9%). Al Madinah province reported the first mortality in March, and the peak rate was found in June (1.6% of mortality). The first death from COVID-19 was reported in April in the Al Qassim province, and the highest mortality rate was found in August (mortality rate of 1.8%).

Hail province reported the first mortality in June, and the peak rate was found in August (mortality rate 1.5%). The first death in the Northern border province was reported in May and became the highest in August (total mortality rate of 2.4%). The Asir province reported the first mortality in April, but in May, no deaths due to COVID-19 were found. However, in the month of September, the highest rate of mortality was recorded (1.4 total mortality). In Al Baha province, the first death due to COVID-19 was recorded in June, and the peak rate was found in September (total mortality rate of 2.3%), while in Jazan, the first death was reported in April and the peak was found in September (total mortality of 2.1%). Al Jawf (2.5% mortality) and Najran (1.1% mortality) provinces reported the first death due to COVID-19 in June, but the peak mortality was found in July and October, respectively. Tabuk province recorded the first mortality in April but none in May, and the maximum deaths were reported in July (total mortality rate 1.8%).

### 3.6. Case-Fatality Ratio in Different Provinces of Saudi Arabia

**Figure 6:**
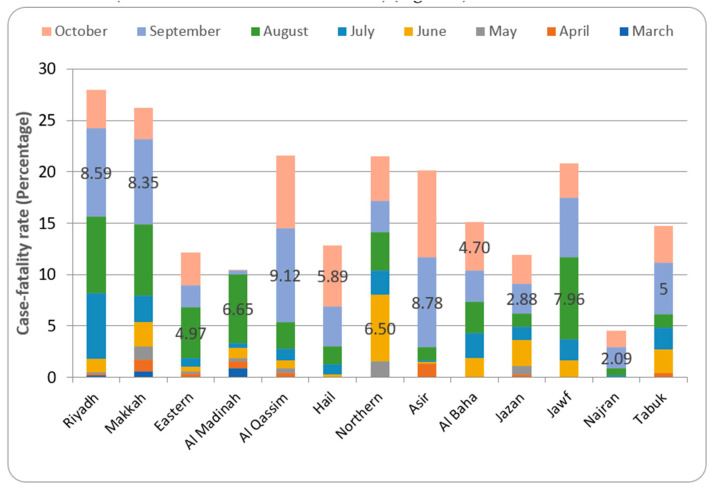
Case–fatality rate in different provinces of Saudi Arabia.

The case–fatality rate suggests that in the month of March, among the three provinces that recorded the deaths (Riyadh—0.17%, Makkah—0.58%, and Al Madinah—0.89%), the highest number was found in Al Madinah province. The peak case–fatality rates observed for different provinces were in June (Northern borders – 6.50%), August (Eastern—4.97%, Al Madinah—6.65% and Jawf—7.96%), September (Riyadh—8.59%, Makkah—8.35%, Al Qassim—9.12, Asir—8.78%, Jazan—2.88%, Najran—2.09% and Tabuk—5.0%), and October (Hail—5.89% and Al Baha—4.70%) (Figure 6).

### 3.7. Total Pattern of Spread in All the Provinces

**Figure 7:**
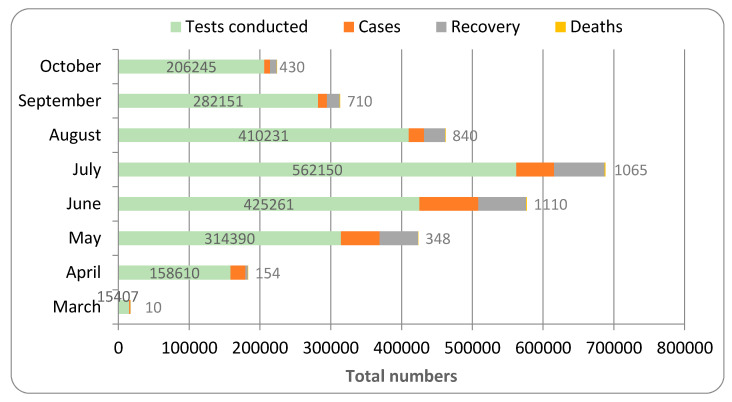
Total pattern of COVID-19 spread in different provinces of Saudi.

Figure 7 depicts the overall pattern of COVID-19 distributed in Saudi Arabia’s various provinces. The number of positive cases started to grow in March 2020 and steadily increased until hitting a peak in June 2020, after which the number of positive cases began to decrease. In July 2020, the rate of recovery was found to be at its peak, and mortality was found to be at its peak in June 2020. Similarly, the total number of tests performed to detect COVID-19 peaked in July and then declined as the number of confirmed cases decreased.

## 4. Discussion

The present study analyzed the pattern of COVID-19 spread in different provinces of Saudi Arabia. There are thirteen provinces comprised of several cities and small towns spread across a vast desert region. The data from two to three major cities from each province were recorded from the official website of the Saudi Ministry of Health on an Excel sheet, which was then analyzed graphically and represented in Figure 1, Figure 2, Figure 3, Figure 4 and Figure 5.

The pattern of spread of the infection in most of the provinces in Saudi Arabia indicated that the disease was detected in the month of March and the peak rate of infection was found in the months of June and July. RT-PCR swab testing is performed in Saudi Arabia since this test is reported to be accurate and efficient, and it detects COVID-19 infection even before the person becomes infectious (https://www.moh.gov.sa, accessed 12 June 2021). The total number of tests performed for both symptomatic and asymptomatic people is represented in Figure 7. The testing of suspected individuals started at the beginning of 2020, but the first confirmed COVID-19 case was detected in March. The rate of testing increased dramatically as the number of confirmed cases increased, reaching a peak in July. The highest percentage of confirmed COVID-19 cases in comparison to the total number of tests was observed in June (19.55%) (Figure 2). As the number of confirmed cases continues to rise, healthcare authorities increased the testing rate in accordance with international guidelines (6). The rate of infection then showed a progressive decline. However, in a few provinces, such as Al Baha and Jazaan, the peak level of the spread of infection was observed in the months of September and August, respectively (Figure 1, Figure 2 and Figure 3).

COVID-19 is a member of the coronavirus family, which is found throughout the world in various species. Some of the members of this virus have been found to be specific to particular species of animals and have not been shown to cause infection in other species. COVID-19 was found to be common in bats and has been known to be present in them for years. Unless there is a rare case of mutation, these viruses do not normally change hosts [18]. The virus was suspected to have an intermediate host before being shown to infect humans. Although the first reported case of novel coronavirus was from the Wuhan province of China, traces of coronavirus genetic material were found in human samples at a much earlier date [19].

The World Health Organization gave several amendments to describe the pattern of spread of the infection after declaring it a ‘pandemic’. The latest information from the world’s premier health organization is that the coronavirus infection is airborne and spreads by the inhalation of infected droplets coming through coughing, sneezing, speech, and exhaled air [1].

The observations from this study indicated that COVID-19 infection was more prevalent in urbanized regions. The detection and spread of COVID-19 were also found to be early and fast in the major cities of the country (Figure 1, Figure 2, Figure 3, Figure 4, Figure 5, Figure 6 and Figure 7). The pattern of spread of COVID-19 infection indicated that all the provinces of Saudi Arabia except the Northern border and Al Jawf recorded COVID-19 in the month of March (Figure S1). The thickly populated provinces, such as Al Riyadh (0.404% of population), Makkah (0.348% of population), and the East (0.485% of population), recorded their peak in the month of June, except for Al Madinah province, where the highest numbers (0.344% of population) were recorded in May. The moderate to low population regions, such as Al Qasim (0.227%), Hail (0.368%), Aseer (0.274%), Najran (0.472%), the Northern border (0.186%), and Tabuk (0.172%), observed peak cases in the month of July, while Jazan (0.165%) and Al Baha (0.098%) provinces recorded peaks in August and September, respectively. Further, all the provinces showed a decline in the number of COVID-19 cases from the month of October (Figure 1, Figure 2 and Figure 3). The infection rate in different provinces suggested that the thickly populated provinces such as Riyadh, Makkah, and Eastern peaked in the month of June. Moderately populated provinces such as Al Qassim, Hail, Asir, Najran, and Tabuk peaked in July. Al Baha, a less populous but more popular province, peaked in September (Figure 3).

According to a study conducted in this region, men (71.7%) are reported to be more affected by COVID-19 infection compared to women (28.3%). The infection rate in the population was found to be highest in the 30–39 year age group (29.3%), followed by the 20–29 year age group (20.9%), 40–49 year age group (18.4%), 50–59 year age group (11.1%), 10–19 year age group (7.0%), 0–9 year age group (6.2%), 60–69 year age group (4.7%), 70–79 year age group (1.7%), and the lthe80 year age group (0.7%). The reason for this was reported to be the lack of strict precautionary measures followed by the younger generation of the population [20]. Earlier studies indicated that the average age of the Saudi population is 31.8 years. The mean age of the people infected with COVID-19 is 36 years, and deaths have been found to be in patients above 65 years old [21,22]. A study conducted in five major cities in Saudi Arabia indicated that the number of COVID-19 cases increased when the temperature, humidity, and wind speed decreased. Such a type of climate in this region can be seen in the peak winter (December–January) and peak summer (June–August) seasons [13]. In comparison to other Gulf countries (Figure S2), Saudi Arabia has shown an upward trend in infection, while other countries have shown a flattening of the curve since June despite the fact that all countries recorded an infection in March (https://www.moh.gov.sa, accessed on 12 June 2021). Several factors have been linked to this, including a dense human population, crowded areas, frequent human interactions, natural encroachment, inequalities between different classes of society toward the pandemic, and the population’s unhealthy lifestyle, which has resulted in cities becoming the epicenter of the pandemic [23].

The data also showed that the provinces with the highest rate of infection also had the highest rate of recovery, either in the same month or the month after (Figure 1, Figure 2, Figure 3, Figure 4, Figure 5, Figure 6 and Figure 7). The rate of recovery in the kingdom has now reached close to 97%. Several factors have been attributed to the recovery from the COVID-19 infection. The first and most important factor is the extent of the viral load carried by the susceptible individual. The compulsion to use a facemask, frequent hand sanitization, and maintaining social distancing have all been effectively implemented by the government authorities (https://www.moh.gov.sa, accessed on 12 June 2021).

These measures have also been advocated by the WHO as preventive strategies to contain the spread of infections in the population. Previous research has shown that if the virus load is limited, people may become ill, but the immune response effectively fights off the infection [24]. Together, the awareness of health providers created through various media in the public has significantly contributed to seeking medical interventions whenever there are symptoms. During the COVID-19 pandemic, the whole world worked as a single unit, where medical knowledge was freely exchanged between nations, including the Arabian peninsula countries. The cooperation of the healthcare organizations of different countries to share information for the treatment of COVID-19, such as the specific use of corticosteroids, anticoagulants, and antiviral agents and the duration between vaccine doses, has also helped in effectively treating the specific complications of the infection. Additionally, WHO regularly updates all the new approaches to the treatment and management of COVID-19 on its official website [25].

The case–fatality ratio (CFR) indicated that different provinces of the country peaked in different months. Most of the provinces showed a peak CFR in the month of September (Figure 6). In Saudi Arabia, September is the month when the peak of summer ends, and it is associated with dust storms. This could have complicated the existing respiratory ailments in the patients, leading to higher CFRs (13, 20, 21). Additionally, the current study found that the mortality rate in the Kingdom is around 1–2% (Figure 5 and Figure 6), which is considered to be within the average found in countries that effectively deal with the infection, such as Switzerland, South Korea, and New Zealand. COVID-19 mortality is determined by a number of factors. The most important thing is the comorbid conditions of the patient. The prevalence of existing respiratory and cardiovascular diseases is reported to be the important determinant of the occurrence of complications of COVID-19 infection [26].

According to the WHO, the pandemic situation is still prevalent in major parts of the globe. It is essential for all healthcare members to work unitedly to defeat COVID-19. The precautionary and preventive measures being implemented by the government agencies in the Kingdom of Saudi Arabia include a blanket ban on international travel, especially to the countries severely affected by COVID-19. People coming from permitted countries are required to undergo quarantine and compulsory PCR tests before being allowed free movement in the country. Precautionary measures are strictly implemented in public places such as prayer halls, malls, hospitals, universities, and marketplaces. Online classes for school-going children have been made compulsory. Prior permission is mandatory for private functions where people gather. Wearing masks and maintaining social distancing are strictly followed in all public places. Mass immunization of the general public including children of age 12 to 18 years is performed free of cost and is taking place in all provinces at a war-footing level. Further, separate medical facilities are provided for COVID-19 patients as well as those who might have come into contact with a patient (https://www.moh.gov.sa, accessed on 13 July 2021).

Due to the above-mentioned measures, currently, the rate of COVID-19 infection is showing a progressive decline in different provinces of Saudi Arabia. This is one of the remarkable accomplishments at a time when infection rates are rising in many other countries. According to official data, about 70% of the adult population is covered with the first dose of the COVID vaccine, and the second dose is underway in all the regions of the country. The proactive measures taken by the government agencies in the Kingdom of Saudi Arabia are attributed to the effective control of infection and must be continued until the end of the pandemic. The health status of the public, including the infected and immunized, is being recorded in a common platform, and restriction of entry is being planned for non-immunized individuals in public places. As per the WHO, there is a need to defeat the pandemic. It has suggested the immunization of every individual in the world and urged every country to cooperate in its mission. It has suggested elaborate research and a necessity to find effective therapies, including vaccines, to prevent future occurrences of pandemics [25]. Additionally, the government authorities regularly update and follow international guidelines for the effective control of pandemic situations such as COVID-19 (https://www.moh.gov.sa. Accessed on 13 July 2021). In addition, the country supports local and international research for the development of new medical interventions to treat global health issues including coronavirus infections [14,21].

## 5. Conclusions

According to this report, the pattern of COVID-19 spread in most provinces began in March and peaked in June and July. The death rate was estimated to be around 1–2%. Despite the fact that the COVID-19 infection is declining, it is still present in all provinces. To fight the COVID-19 pandemic, precautionary steps and successful treatment methods, such as vaccination, are considered the most suitable strategies.

## Data Availability

Data are contained within the article.

## Supplementary Materials

The following are available online at https://www.mdpi.com/article/10.3390/healthcare9080931/s1: Figure S1: Geographical distribution of provinces of Saudi Arabia, Figure S2: Number of COVID-19 infection in gulf countries.
