# Supplementary material for: Infection Spread, Recovery, and Fatality from Coronavirus in Different Provinces of Saudi Arabia
Source: Healthcare (Basel). 2021 Jul 24;9(8):931. doi: 10.3390/healthcare9080931 (PMC8394587; doi:10.3390/healthcare9080931)

Article

Infection spread, recovery and fatality from coronavirus in different provinces of Saudi Arabia

Mohammed Muberek Alharbi[1], Syed Imam Rabbani[1], Syed Mohammed Basheeruddin Asdaq[2*], Abdulhakeem S. Alamri[3,4], Walaa F. Alsanie[3,4], Majid Alhomrani[3,4], Yahya Mohzari[5], Ahmed Alrashed[6], Reem Faisal Bamogaddam[7], Saleh Ahmad Alajlan[6], Mansour A. Alharbi[5], Norah N. Aldhawyan[5], Saeed Y. Najmi[7]

[1]Department of Pharmacology and Toxicology, College of Pharmacy, Qassim University, Buraydah, Kingdom of Saudi Arabia, syedrabbani09@yahoo.com (S.I.R.);mhospital1920@gmail.com (M.M.A)

[2*]Department of Pharmacy Practice, College of Pharmacy, AlMaarefa University, Dariyah, 13713, Riyadh, Saudi Arabia, sasdaq@gmail.com

[3]Department of Clinical Laboratory Sciences, The Faculty of Applied Medical Sciences, Taif University, Taif, Saudi Arabia, a.alamri@tu.edu.sa (A.S.A), w.alsanie@tu.edu.sa (W.F.A), m.alhomrani@tu.edu.sa (M.A)

[4]Centre of Biomedical Sciences Research (CBSR), Deanship of Scientific Research, Taif University, Saudi Arabia

[5]Clinical pharmacy department, King Saud Medical City, Riyadh, Saudi Arabia 12746, Yali2016@hotmail.com (Y.M); manalharbi87@gmail.com (M.A.A); Ph.norahnd@gmail.com (N.A.A)

[6]Pharmaceutical services administration, Inpatient Department, main hospital, KFMC, Riyadh, Saudi Arabia, 11564, emadasdaq@gmail.com (A.A) emadfaiqa@gmail.com (S.A.A)

[7]Pharmaceutical Services Department, Clinical Pharmacy, King Saud Medical City, Riyadh, Saudi Arabia, farhana.basheer13@gmail.com

[6]Pharmacy Administration, King Fahad Medical City, Riyadh, Saudi Arabia, emadfaiqa@gmail.com (A.S.A); qualityasdaq@gmail.com (S.A.A)

[8]Pharmaceutical Care Services, Samtah General Services, Jazan, Ministry of Health, Saudi Arabia: sanajmi-2017@hotmail.com

*Correspondence: sasdaq@gmail.com; Tel.: +966-1-403555-3399

Supplementary figures:

Figure S1: Geographical distribution of provinces of Saudi Arabia

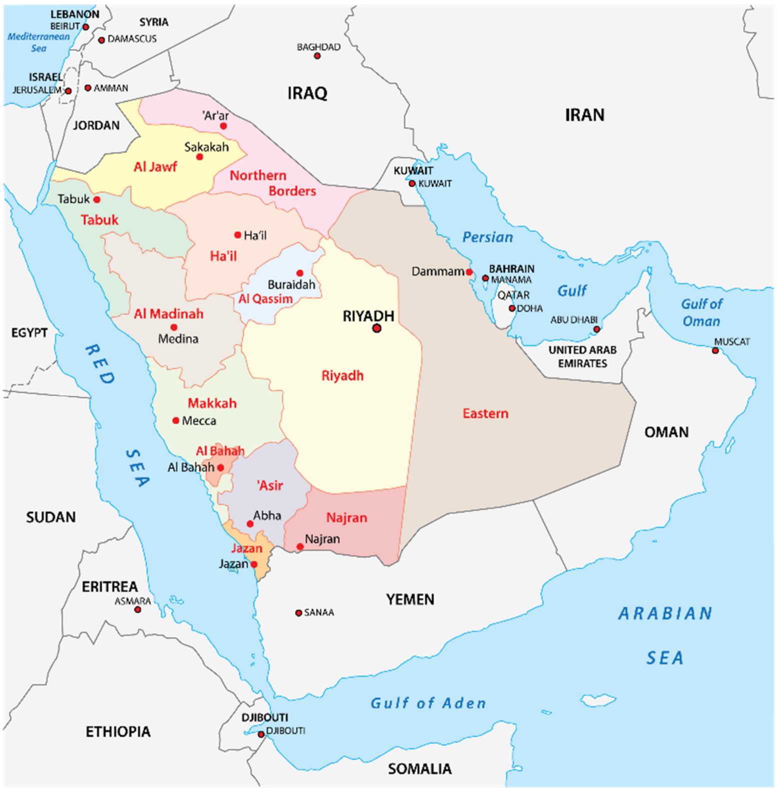

1.

[Riyadh – 6505509 (24.9%), Makkah – 6662597 (25.5%), Al Madhinah – 1694749 (6.5%), Al Qassim – 1184365 (4.5%), Eastern – 379973 (14.6), Aseer – 1897236 (7.3%), Tabuk – 777680 (7.3%), Hail – 593308 (2.3%), Northern Borders – 311473 (1.2%), Jazan – 1332262 (5.1%), Najran – 496613 (1.9%), Al Baha – 406724 (1.6%) and Al Jowf – 428266 (1.6%)]

2. Figure S2: Number of COVID19 infection in gulf countries

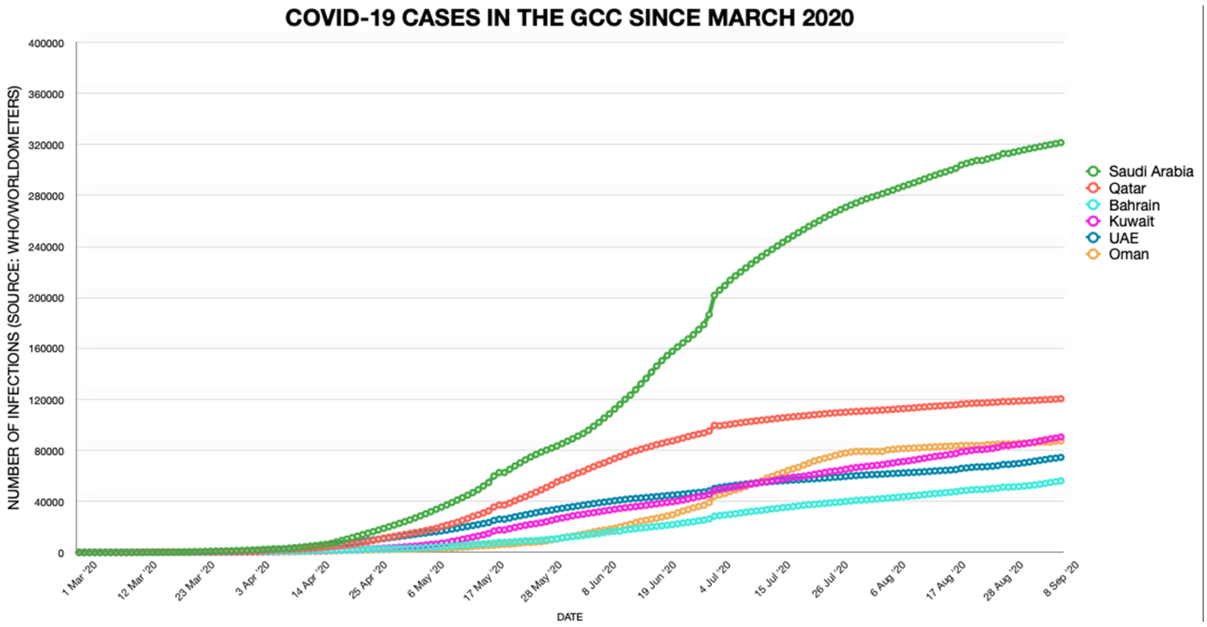

Supplement: Supplementary file 1 [file healthcare-09-00931-s001.zip › healthcare-1234655-SI.pdf]
